# Structural Analysis of the Newly Prepared Ti55Al27Mo13 Alloy by Aluminothermic Reaction

**DOI:** 10.3390/ma18153583

**Published:** 2025-07-30

**Authors:** Štefan Michna, Jaroslava Svobodová, Anna Knaislová, Jan Novotný, Lenka Michnová

**Affiliations:** 1 Faculty of Mechanical Engineering, J. E. Purkyne Universty in Usti nad Labem, Pasteurova 3334/7, 400 96 Usti nad Labem, Czech Republic; stefan.michna@ujep.cz (Š.M.); anna.knaislova@ujep.cz (A.K.); jan.novotny@ujep.cz (J.N.);; 2 Faculty of Engineering, Czech University of Life Sciences, Kamýcká 129, 165 00 Prague–Suchdol, Czech Republic; 3 Faculty of Environment, J. E. Purkyne Universty in Usti nad Labem, Pasteurova 3632/15, 400 96 Usti nad Labem, Czech Republic

**Keywords:** aluminothermic reaction, Ti–Al–Mo alloy, chemical composition, scanning electron microscopy, EDS analysis

## Abstract

This study presents the structural and compositional characterisation of a newly developed Ti55Al27Mo13 alloy synthesised via aluminothermic reaction. The alloy was designed to overcome the limitations of conventional processing routes for high–melting–point elements such as Ti and Mo, enabling the formation of a complex, multi–phase microstructure in a single high–temperature step. The aim was to develop and characterise a material with microstructural features expected to enhance wear resistance, oxidation behaviour, and thermal stability in future applications. The alloy is intended as a precursor for composite nanopowders and surface coatings applied to aluminium–, magnesium–, and iron–based substrates subjected to mechanical and thermal loading. Elemental analysis (XRF, EDS) confirmed the presence of Ti, Al, Mo, and minor elements such as Si, Fe, and C. Microstructural investigations using laser confocal and scanning electron microscopy revealed a heterogeneous structure comprising solid solutions, eutectic regions, and dispersed oxide and carbide phases. Notably, the alloy exhibits high hardness values, reaching >2400 HV in Al_2_O_3_ regions and ~1300 HV in Mo– and Si–enriched solid solutions. These results suggest the material’s substantial potential for protective surface engineering. Further tribological, thermal, and corrosion testing, conducted with meticulous attention to detail, will follow to validate its functional performance in target applications.

## 1. Introduction

The development of advanced multi–component alloys such as Ti–Al–Mo is motivated by the demand for materials that exhibit a unique combination of low density, high strength at elevated temperatures, excellent wear resistance, and oxidation stability. These properties are especially valuable in high–performance applications such as aerospace, automotive, and tooling industries. However, conventional alloying methods face substantial challenges when combining elements with significantly different melting points—e.g., aluminium (660 °C), titanium (1668 °C), and molybdenum (2622 °C). In addition, the differences in density and limited mutual solubility of these elements often result in phase segregation or poor microstructural homogeneity. In preparing these multi–component alloys containing hard–to–melt metals, such as aluminium, the problem is also that they do not form solid solutions with each other in most cases. Conventional methods are hardly capable of preparing such alloys [1,2,3].

To overcome these limitations, the aluminothermic reaction presents a viable route for alloy synthesis. It is a strongly exothermic reduction process in which aluminium acts as a reducing agent for metal oxides, generating temperatures up to 2500–3000 °C. This makes it particularly practical and applicable for the preparation of alloys containing high–melting–point elements such as Mo and Ti. Aluminothermic synthesis also enables the in situ formation of hard ceramic or carbide phases (e.g., Al_2_O_3_, TiC, MoC, Ti_2_AlC), further enhancing the mechanical and thermal properties of the final material.

The principle of the aluminothermic reaction consists of a strongly exothermic reaction that uses powdered aluminium as a reducing agent at high temperatures in a very short time. This process is used, for example, in the production of iron alloys. The reaction mechanism involves the high affinity of aluminium for oxygen, resulting in the formation of stable Al_2_O_3_ (aluminium oxide) [2,3,4,5,6,7]. The general equation can be expressed as follows:

Al + XO → Al_2_O_3_ + X + Q (1)

where Al—aluminium; XO—metal oxide, non–metal oxide; X—metal, non–metal; Q—heat generated during the reaction (energy). Any oxide with a Gibbs free energy of formation higher than that of Al_2_O_3_ can be used as the reactant oxide (XO) [2,3,4,5,6,7].

This reaction can proceed even in oxygen–free or underwater environments due to its self–sustaining oxygen balance [8,9]. In refractory metals such as chromium, titanium, or tungsten, it is necessary to thermally stimulate the process, which can be achieved using thermite. Thermite is a pyrotechnic mixture that burns at very high temperatures. It is most commonly composed of pyroaluminium (approx. 25 wt.%) and iron (II, III) oxide (approx. 75 wt.%). Using this mixture, temperatures of up to 2500 °C can be reached [10]. Aluminothermic reactions of various metal oxides (e.g., TiO_2_, SiO_2_, ZnO, Cr_2_O_3_, CuO) have been studied for many years [1]. These reactions are useful in the production of metals and alloys, such as refractory ceramics, composite materials, nanowires and nano–coatings, ceramic coatings on metallic pipes, and in railway welding [2,11,12,13].

The burning of thermite is based on an aluminothermic reaction in a reducing system, where the reducing agent is powdered aluminium and the oxidising agent is metal oxide, according to the response at very high temperature [14,15]:

Fe_2_O_3_ + 2Al → Al_2_O_3_ + 2Fe + Q (2)

The magnetic oxide of iron may also be used in the thermit mixture. It reacts as follows:

3Fe_3_O_4_ + 8Al → 4Al_2_O_3_ + 9Fe + Q (3)

One specific process in the aluminothermic production of alloys is the recovery of part of the refractory metals (Mo, Cr, W, Co, Ti) in the form of metal carbides. In this case, the aluminothermic reaction is used deliberately, where the mixture added to the carbon crucible consists of individual powder components (MoO_3_, WO_3_, Co_2_O_3_, coal, aluminium, etc.) according to the desired final composition. The mixture is heated in an electric furnace at 900 °C in an argon stream, and a brief, rapid heating completes the synthesis to 1000 °C (ignition for the aluminothermic reaction). After cooling, the obtained material is ground and sieved to the required particle size of about 1 µm [16,17,18,19].

In this context, a novel Ti55Al27Mo13 alloy was meticulously designed and prepared using aluminothermic reduction. The aim was to create a material with a unique microstructure and performance, intended for the subsequent preparation of composite nanopowders and advanced surface coatings for aluminium–, magnesium–, and iron–based substrates. The heterogeneous microstructure, comprising solid solutions, eutectic phases, and ceramic particles, is anticipated to deliver high hardness, wear resistance, and improved thermal stability. The inclusion of alloying elements, such as silicon, further enhances the alloy’s properties, promoting the formation of dense, protective oxide layers and contributing to its oxidation resistance. This study presents the structural, compositional, and microstructural characterization of the Ti55Al27Mo13 alloy, discusses the effects of its constituent elements, and compares its properties with those of selected high–performance titanium alloys.

The newly designed Ti55Al27Mo13 alloy was specifically developed to serve as a precursor for composite nanopowders and wear–resistant surface coatings, opening up exciting possibilities for the future of materials science. The presence of high–melting elements and the resulting heterogeneous microstructure, including hard dispersed phases and solid solutions, enhances the alloy’s mechanical and thermal properties, making it a promising candidate for surface functionalization of aluminium–, magnesium–, and iron–based components exposed to aggressive environments. The objective was not only to explore the potential of the Ti–Al–Mo system in non–conventional synthesis routes but also to develop a multifunctional material with tailored performance for coating technologies. Although Ti–Al–Mo alloys are known for their high–temperature strength and oxidation resistance, the specific Ti55Al27Mo13 composition developed in this work is novel in terms of its synthesis route and resulting phase structure. The alloy was prepared by aluminothermic reduction, which enabled the direct formation of a complex, multiphase microstructure, including challenging ceramic phases, in a single high–temperature step. This approach differs significantly from conventional melting or powder metallurgy techniques applied to similar compositions.

In this study, a new Ti55Al27Mo13 alloy was developed using aluminothermic reduction. This unconventional synthesis route enables the in situ formation of challenging ceramic phases, such as Al_2_O_3_ and TiC, during alloy solidification. The key innovation lies in combining a novel composition with a cost– and energy–effective one–step preparation method, resulting in a complex multiphase structure with high localised hardness. The primary objectives of this research were (i) to design and prepare the Ti–Al–Mo alloy via aluminothermic reaction, (ii) to perform detailed structural and microstructural characterization using XRF, EDS, laser confocal and electron microscopy, and (iii) to assess the phase–specific mechanical properties using microhardness measurements. The findings provide a foundation for the alloy’s future use as a precursor material for surface coatings and composite powders in wear– and heat–resistant applications.

### 1.1. Ternary System Ti–Al–Mo

The Ti–Al–Mo ternary system has attracted considerable attention due to its critical role in the development of advanced titanium aluminide–based alloys. These alloys are renowned for their exceptional high–temperature strength, low density, good oxidation resistance, and potential for use in structural applications across the aerospace and automotive industries. However, limitations such as poor room–temperature ductility have driven the need for alloying strategies that can enhance mechanical performance [20,21,22,23,24].

Molybdenum (Mo) is a practical alloying element in TiAl–based systems. It acts as a β–phase stabiliser, promoting the formation of the ductile β(Ti, Mo) phase and significantly improving creep resistance due to its low diffusivity in this phase. Experimental studies on the Ti–Al–Mo phase equilibria, such as those by Huang et al. [24], have provided essential insights into the phase stability and interactions within this system, which are particularly relevant when analysing new alloys such as Ti55Al27Mo13.

The phase equilibria in the Ti–Al–Mo system were established at multiple temperatures (1073–1473 K), revealing that the β(Ti, Mo), TiAl, TiAl_3_, and AlMo_3_ phases exhibit broad compositional ranges. Specifically, the solubility of Al in β(Ti, Mo) decreases with temperature, from 49.0 at.% at 1 473 K to 39.9 at.% at 1073 K—while Ti solubility in AlMo_3_ declines from 25.4 at.% to 20.8 at.% across the same temperature range. These wide solubility ranges indicate the flexibility of this system for tailoring alloy compositions.

Of particular interest is the formation of the ternary ρ–phase (r phase), which arises through a peritectoid reaction (β(Ti, Mo) + AlMo_3_ → ρ) between 1373 and 1423 K. This phase is also involved in other multi–phase equilibria at lower temperatures, including peri–eutectoid and invariant reactions such as

β(Ti,Mo) + AlMo_3_ + ρ + TiAl_3_ (1273–1373 K) (4)

β(Ti,Mo) + TiAl_3_ + ρ + TiAl (1173–1273 K) (5)

#### 1.1.1. Effect of Mo on the Properties and Structure of Ti–Al

**Figure 1:**
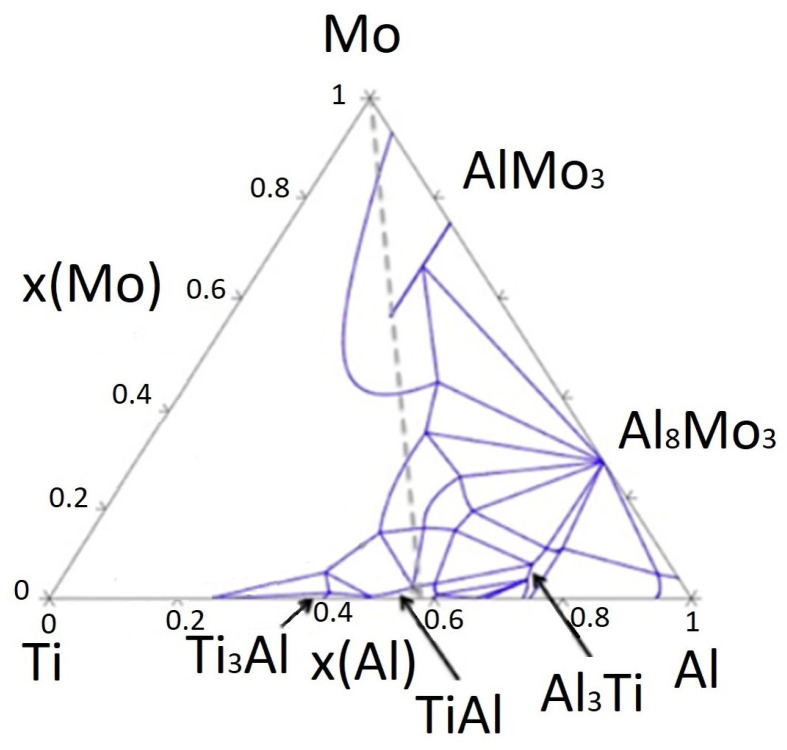
Ti–Al–Mo ternary diagram [25].

In Ti–Al alloys, the primary phases present are the γ (TiAl) phase, the αTi phase (hexagonal close–packed), and the βTi phase (body–centred cubic). The addition of Mo, as a β–stabiliser, promotes the formation of the βTi phase, which enhances the mechanical and thermal stability of the alloy. Mo increases the strength and hardness of the alloys by stabilising the high–temperature β phase and refining the microstructure. According to Kulkarni [25], the isothermal section at 1200 °C shows that the solubility of Al in β(Ti, Mo) reaches ~45 at.% and that the β + TiAl + TiAl_3_ phase field predominates at this temperature, which aligns with our microstructural observations. This diagram illustrates the phase fields and diffusion pathways within the Ti–Al–Mo system, highlighting the β–phase field and multiphase regions (see Figure 1).

The phase transformations in Ti–Al–Mo alloys depend on both the Mo content and the heat treatment processes [20,24,26,27,28,29,30,31]. For instance, small additions of Mo (1–2 at.%) have been shown to promote a eutectoid transformation, leading to a fine lamellar structure, which improves the balance between strength and ductility. With increasing Mo content (more than 6 at.%), the βTi phase becomes the dominant phase, resulting in a transition from a lamellar or dendritic microstructure to one dominated by β grains. This modification in the microstructure directly affects the alloy’s mechanical properties, including toughness, strength, and resistance to high–temperature oxidation [20,26,28,29,30].

In particular, the martensitic transformation, which is common in Ti–Al alloys, can be influenced by the addition of Mo. At higher Mo concentrations (e.g., 8 at.%), the presence of fine martensitic needles near the grain boundaries can further enhance strength. The formation of Mo–rich phases, particularly at the boundaries of eutectoid grains, enhances the alloy’s thermal and mechanical stability, enabling the material to perform well under extreme conditions (20,28,29]. Theoretical studies and phase diagrams of the Ti–Al–Mo system suggest that Mo addition can shift phase boundaries and alter the solidification path of the alloy [27]. This influences the alloy’s phase composition during cooling and solidification, favouring either peritectic or eutectoid transformations depending on the Mo content. The β → α + γ transformation is crucial in controlling the overall mechanical properties, with Mo slowing down or stabilising specific phase transitions to improve performance.

At higher Mo concentrations, such as 12 at. or above this percentage, the βTi phase remains stable across a broader range of temperatures. This β–phase stability suppresses the formation of αTi and γ phases, resulting in a microstructure dominated by β grains that are coarser and more uniformly distributed. This results in improved high–temperature creep resistance and enhanced oxidation resistance—the addition of more than 12 at.% Mo significantly alters the solidification behaviour of the Ti–Al–Mo alloy. As indicated in the Ti–Al–Mo phase diagram, increasing the Mo content shifts the solidification path towards the stabilisation of the β–Ti phase at lower temperatures. The typical solidification sequence transitions from L → βTi → α + γ (at lower Mo content) to a peritectic transformation at higher Mo concentrations, where residual βTi remains unconsumed by the peritectic process and forms Mo–rich zones within the microstructure. These Mo–rich β–Ti phases serve as sites for grain boundary strengthening and enhancing thermal stability.

At very high Mo concentrations, a martensitic transformation can also be suppressed, resulting in fewer martensitic needles or even their complete absence, as the βTi phase remains stable throughout cooling. This phase stabilisation plays a key role in preventing brittle fracture, enhancing the alloy’s fracture toughness, and allowing for greater workability during manufacturing processes [22,24,29]. Yang et al. [26] demonstrated that increasing Al and decreasing Mo in TNM (TiAl–based intermetallic alloys containing Nb and Mo) alloys expands lamellar spacing and enhances the volume fraction of the β_0_ phase. These effects align with the trends observed in our as–cast Ti55Al27Mo13 alloy.

#### 1.1.2. Effect of Si on Properties of Ti–Al or Ti–Al–Mo Alloy

Silicon is an admixture from the process of preparing the alloy by aluminothermic melting in the Ti–Al–Mo alloy, and its content reaches approximately 2.0 at.%, which represents most of the admixtures present in the produced alloy. Generally, as the silicon content in the alloy increases, the hardness of these alloys increases with the volume fraction of silicide Ti_5_Si_3_, reaching a value of almost 800 HV. Also, the oxidation resistance of the prepared Ti–Al–Si alloys at temperatures of 800–900 °C is higher than that of the binary Ti–Al alloy produced by fusion metallurgy processes. When these materials are used in powder form for surface coating, the positive effect of silicon on the compactness and adhesion of the oxide layer is manifested by the formation of silicon dioxide, which fills the pores in the oxide layer formed by a mixture of TiO_2_ (rutile) and Al_2_O_3_. The resistance of Ti–Al–Si materials to high–temperature oxidation is approximately comparable to that of commercial nickel alloys designed for high–temperature applications. Compared to titanium of metallurgical purity, these materials have an oxidation rate up to a hundred times lower at temperatures of 800–900 °C [32,33,34,35].

## 2. Materials and Methods

A specially designed multicomponent alloy with a nominal composition of Ti55Al27Mo13 (at.%) was synthesised using an aluminothermic reduction process. The nominal composition of Ti55Al27Mo13 was achieved by precisely weighing and mixing the oxide precursors and aluminium powder, guided by stoichiometric calculations. Adjustments were made to account for the purity of reagents and potential processing losses. The detailed synthesis protocol and materials list are provided to ensure reproducibility.

**Figure 2:**
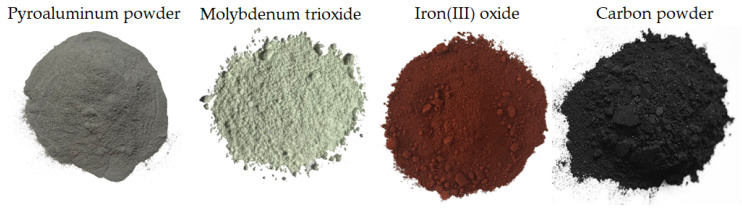
Powders for Ti55Al27Mo13 alloy preparation.

High–purity powdered raw materials, selected for their quality and consistency, were used and weighed to achieve the target composition. The batch consisted of the following (Table 1, Figure 2):

- Pyroaluminium powder (99.8% purity, particle size 0.04–0.1 mm),
- Iron(III) oxide (α–phase, natural hematite, brownish–red powder, particle size ≤1 mm),
- Molybdenum trioxide (MoO_3_) (99.5% purity, particle size 1–4 mm),
- Titanium dioxide (TiO_2_) (≥98.0% purity, particle size ≤2 mm),
- Carbon powder (≥98.0% purity, derived from coke or anthracite).

The thermite mixture, composed of powdered aluminium and iron oxide, was adjusted to a 1.2:3 Al–Fe_2_O_3_ mass ratio (240 g pyroaluminium and 600 g Fe_2_O_3_) to ensure a higher residual Al content in the final alloy (targeting 20–30 at.% Al). To support the exothermic reaction and protect the melt from oxidation, 20 g of a slag–forming additive (natural calcium fluoride, CaF_2_—fluorite) was added. This compound formed a floating slag layer that was separated after solidification. Its function was to protect the melt from the atmosphere and to bind oxides and impurities from the raw materials. In addition, 200 g of MoO_3_ and 600 g of TiO_2_ were added to the batch. The charge was placed into a large graphite crucible and inserted into an electric resistance furnace. The batch was gradually heated to ~1000 °C, which is sufficient to trigger the thermite reaction. Notably, the furnace served only for preheating; the aluminothermic reaction itself, with its impressive self–sustaining nature, begins spontaneously once the ignition temperature is reached (~850–900 °C). After ignition, the reaction proceeds vigorously without further heating, with internal temperatures reaching up to 2000–3000 °C for several minutes. These high reaction temperatures result from the exothermic enthalpy of aluminium reacting with metal oxides and are not externally controlled.

**Figure 3:**
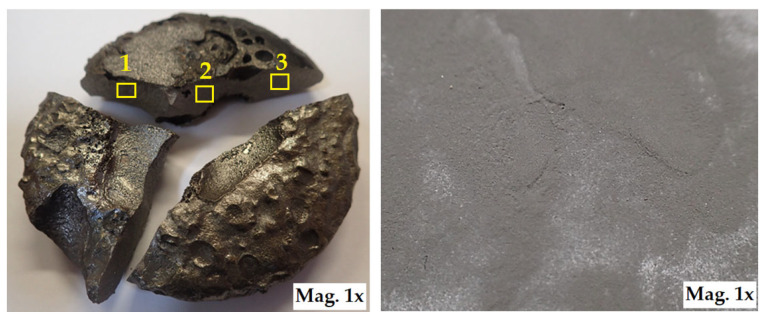
Ti55Al27Mo13 alloy after aluminothermic melting, and nanopowder after crushing and milling, areas 1, 2, 3 indicate sampling locations for SEM micrographs.

The aluminothermic reduction mechanism hinges on the unique properties of aluminium, which acts as a potent reducing agent due to its high affinity for oxygen. In the classic thermite reaction, aluminium serves as the reductant, while the metal oxides (e.g., Fe_2_O_3_, MoO_3_, TiO_2_) function as oxidants. This reduction process results in the formation of metallic phases (Ti, Mo) and the simultaneous creation of in situ ceramic particles such as Al_2_O_3_ and TiC, due to the reaction of reduced metals with the carbon present in the batch. The molten metal and slag separate by density, and after cooling, the alloy ingot is extracted from the bottom of the crucible. This forms a compact layer of alloy free from slag (Figure 3). After cooling, the brittle slag, which remains on top, can be easily separated from the compact and rugged composite alloy. Following cleaning, the alloy is first crushed and then ground in a planetary mill to produce a fine nanopowder for coating applications (Figure 3).

All starting materials, including their purities, particle sizes, and weighed amounts, are meticulously listed. This comprehensive detail is provided to ensure the reproducibility of the process, assuring the reader of the thoroughness and reliability of the research.

The overall metallurgical process of the Ti55Al27Mo13 alloy (composition in wt.%) preparation in the presence of carbon can be expressed by the following chemical reactions:

MoO_3_ + 2Al → Al_2_O_3_ + Mo (6)

3TiO_2_ + 4Al → 2Al_2_O_3_ + 3Ti (7)

The following reaction is also possible:

Fe_2_O_3_ + 2Al → Al_2_O_3_ + 2Fe (8)

Due to the presence of carbon, reactions also occur in part:

3Mo + 2C → Mo_3_C_2_ (9)

Ti + C → TiC(10)

Due to the presence of the elements Mo and Ti, complex carbides form in a certain stoichiometric ratio, such as

Mo + Ti + C → (Mo,Ti)C(11)

One of the shortcomings of the aluminothermic reaction is that metals are obtained from metal oxides that are contaminated with other impurity elements, and their purity is 98.5–99.5%. The purity of the final product (manufactured alloy) is lower. The lack of aluminothermic reaction and the charge composition will affect the elemental content of the alloy. In this case, Ti, Al, and Mo will also contain, in units and tenths of a percent, other impurity elements, e.g., Si, Fe, Mn, Cu, etc. Furthermore, from the perspective of the alloy preparation technology used, it is expected that oxide or carbon residues will be present in the structure in the form of carbides. Trace elements such as Si and Fe were not intentionally added but are considered to originate from impurities in the technical–grade aluminium powder and molybdenum reagent. Carbon was deliberately added in the form of graphite powder to support the formation of challenging carbide phases (TiC) during the aluminothermic reaction. The presence of these minor elements is consistent with the composition of the raw material and the processing environment.

After solidification, we obtained the synthesised Ti55Al27Mo13 alloy as a dense metallic puck (see Figure 3), which was then cut into smaller segments using a diamond cutting disc. These segments we manually crushed into coarse fragments suitable for mechanical milling. The crushed alloy fragments were first subjected to coarse grinding in a Retsch PM100 planetary ball mill (Retsch GmbH, Haan, Germany) using 28 mm diameter steel balls. Subsequently, fine grinding was carried out using 3 mm diameter balls under the same milling conditions. All milling steps were performed in stainless steel bowls. To ensure uniform particle size distribution, the powder was sieved in stages using a set of stainless–steel sieves:

The finest sieve had an aperture size of 20 µm,

- The coarsest sieve had an aperture of 1.0 mm.
- Particles larger than 1 mm were returned to the milling cycle for reprocessing.

The ball–to–powder ratio (BPR) was maintained at 5:1, with 500 g of grinding balls and 100 g of alloy powder per batch. The optimised milling parameters were

- 400 rpm for 5 min during coarse grinding,
- Followed by 400 rpm for 10 min during fine grinding.

To ensure the purity of the alloy and prevent cross–contamination between batches of different compositions, the milling bowls and balls undergo a thorough cleaning process between uses. This meticulous cleaning, performed using crushed glass, effectively removes residual particles without introducing unwanted impurities, thereby maintaining the alloy’s integrity.

All powders used in the alloy synthesis process—including Al, Fe_2_O_3_, MoO_3_, TiO_2_, and C—are mechanically mixed in the dry state before loading into the graphite crucible. This mixture undergoes a manual homogenization process in a polyethene container, where it is gently tumbled for 5–10 min. This meticulous process ensures the uniform distribution of particle sizes and components before the thermite reaction, thereby contributing to the quality of the final alloy.

The selected composition of Ti55Al27Mo13 was not strictly optimised due to the inherent limitations of the aluminothermic reduction process. This technique employs metal oxides of limited purity (typically 98.5–99.5%), which may contain impurities such as Si, Fe, Mn, or Cu. Additionally, the presence of carbon and process–related variables can lead to partial formation of carbides and oxide residues in both the melt and slag. The experimental aim was to synthesise an alloy within the targeted compositional window of Ti 50–60 at.%, Al 20–30 at.%, and Mo 10–15 at.%, which was achieved in the final alloy. Minor concentrations of additional elements, present at levels of tenths to units of at.%, were confirmed by chemical analysis. The formation of complex carbides such as (Mo, Ti)C is thermodynamically expected under these conditions. Therefore, the alloy composition reported herein represents a realistic outcome of the process rather than a precisely optimised stoichiometry.

**Table 1:** Starting materials used for the synthesis of the Ti55Al27Mo13 alloy.

No.	Compound	Chemical Formula	Purity [%]	Particle Size [mm]	Weight [g]	Supplier (Country)
1	Pyroaluminium powder	Al	99.8	0.04–0.10	240	Thermo Fisher Scientific (Waltham, MA, USA)
2	Iron(III) oxide (hematite)	Fe_2_O_3_	–	≤1.00 (natural mineral)	600	Thermo Fisher Scientific (Waltham, MA, USA)
3	Molybdenum trioxide	MoO_3_	99.5	1–4	200	Thermo Fisher Scientific (Waltham, MA, USA)
4	Titanium dioxide	TiO_2_	≥98.0	≤2.00	600	Precheza a.s. (Přerov, Czech Republic)
5	Powdered carbon	C	≥98.0	(fine powder)	–	BorsodChem MCHZ s.r.o. (Ostrava, Czech Republic)
6	Calcium fluoride (slag additive)	CaF_2_	–	Granular (technical grade)	20	BorsodChem MCHZ s.r.o. (Ostrava, Czech Republic)

**Figure 4:**
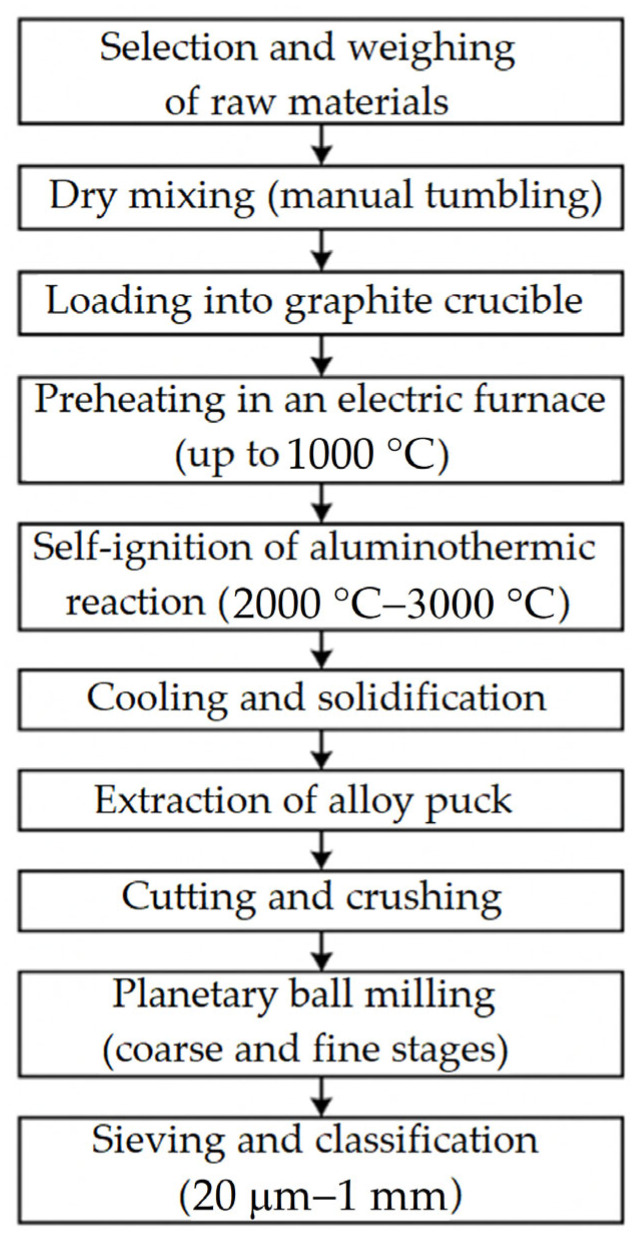
Schematic illustration of the Ti55Al27Mo13 alloy full manufacturing process.

To improve the clarity of the experimental procedure, we have added a schematic illustration (Figure 4) that summarises the whole manufacturing process of the Ti55Al27Mo13 alloy—from the selection of raw materials through aluminothermic reduction, post–processing, and powder preparation. This visual representation will help readers understand the step–by–step workflow of the synthesis route.

An X–ray fluorescence method was used to analyse the chemical composition of the produced Ti55Al27Mo13 alloy; this was used to determine the chemical composition of the material. The measurement was performed using a portable hand–held X–ray spectrometer with a metal analyser DELTA PROFESSIONAL SDD (Silicon Drift Det) (BAS Rudice, Blansko, Czech Republic). The DELTA PROFESSIONAL model uses compact X–rays with a power of 4 W, an optimised anode, and the possibility of a maximum current of up to 200 μA.

The metallographic cut was prepared for the microstructure observation. The sample was cut for the smaller parts; mounted in the resin VersoCit suitable for the cold mounting; ground by P80 to P4000 grinding papers (Hermes Schleifmittel GmbH, Hamburg, Germany); and polished by diamond pastes D3, D1, and D0.7 (diamond particles size 3, 1, and 0.7 μm). The sample was etched by a mixture of 10 mL HNO_3_ + 20 mL HCl + 30 mL H_2_O (it was prepared in our laboratory). The microstructure of the alloy was investigated using a LEXT OLS 5000 laser confocal microscope (Olympus, Shinjuku, Japan) and a scanning electron microscope Tescan Vega 3XMU (Tescan, Brno, Czech Republic) with an Oxford Instruments X–max 80 mm^2^ EDS analyser (Oxford Instruments, HighWycombe, UK). Hardness measurements were performed using an ERNST AT 250X hardness tester (CISAM–ERNST s.r.l., Induno Olona VA, Italy) and. Micro–Vickers Hardness Testing Machine HM–200 (Mitutoyo, Takatsu–ku, Kawasaki, Kanagawa, Japan). Hardness was measured on a polished surface using the Rockwell C test method on an ERNST AT 250X hardness tester. The test was performed with a 150 kgf load applied for 10 s using a diamond cone indenter (Rockwell C scale, HRC). This measurement provided an overview of the bulk hardness of the alloy and allowed comparison with other structural materials. Microhardness testing was carried out using a Micro–Vickers Hardness Testing Machine HM–200. The Vickers method with a 10 g test load (HV 0.01) was applied to determine the hardness of individual microstructural constituents, including solid solutions, eutectic structures, and dispersed oxide or carbide phases. All indentations were performed on carefully polished cross–sections prepared according to standard metallographic procedures. At least 7–10 indentations were made for each region of interest, and average values with standard deviations are reported. The use of both methods was essential due to the alloy’s complex, multiphase microstructure, which required localised mechanical evaluation in addition to bulk–scale assessment.

## 3. Results

### 3.1. Produced Alloy Ti55Al27Mo13 Chemical Composition Analysis

The results of the Ti55Al27Mo13 chemical composition analysis are shown in Table 2. We performed the measurement using X–ray fluorescence (XRF). The method and device we used do not allow for measuring carbon content. Three measurements were performed. An EDS analysis was performed using a scanning electron microscope to determine the carbon content. It can be stated, from the Table 2 results, that the alloy Ti55Al27Mo13 contains the basic elements characteristic for this alloy type, namely Ti 54.86 ± 0.41 wt.%, Al 27.34 ± 0.53 wt.%, and Mo 13.62 ± 0.20 wt.%. From a chemical composition point of view, it can be stated that it is a titanium–aluminium–molybdenum alloy of a specific composition with accompanying elements (Si, Fe, Mn, Cu; see Table 2). We used three different analytical techniques to characterise the chemical composition of the Ti55Al27Mo13 alloy, each providing complementary information. X–ray fluorescence spectroscopy (XRF) determined the average bulk composition of the alloy, with a focus on heavier elements (Table 1). Energy–dispersive spectroscopy (EDS) was subsequently used in two modes: area analysis and local phase–specific analysis (Table 2). At the same time, XRF does not detect light elements such as carbon and oxygen; EDS analysis, on the other hand, allows for the detection of these elements, which are essential for evaluating the presence of carbides and oxides. It is worth noting that the number of identified elements varies depending on the analysed region and microstructural features. Therefore, the data from Table 2 and Table 3 are interpreted in the context of the method used and the measurement focus (bulk vs. local composition).

**Table 2:** Results of the produced Ti55Al27Mo13 alloy chemical composition analysed by an X–ray fluorescence spectrometer.

Chemical Element [wt.%]	Measurement 1	Measurement 2	Measurement 3	Average	Standard Deviation
Ti	54.77	54.40	55.41	54.86	0.41
Al	27.41	27.97	26.66	27.34	0.53
Mo	13.74	13.34	13.79	13.62	0.20
Fe	1.02	1.00	0.96	0.99	0.02
Si	2.03	2.27	2.12	2.14	0.09
Mn	0.31	0.25	0.30	0.28	0.02
Cu	0.34	0.325	0.32	0.32	0.008
Co	0.16	0.17	0.16	0.16	0.004
Nb	0.040	0.036	0.038	0.038	0.001

**Figure 5:**
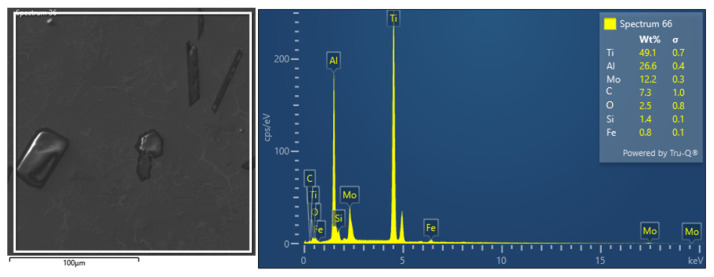
Ti55Al27Mo13 alloy EDS analysis selected area and EDS spectrum of individual elements (area 1 Figure 3).

Area EDS analysis (Figure 5) defined the amount of carbon to be approximately 8 wt.%. It can be stated that the EDS analysis provided semi–quantitative local composition data that were generally consistent with the major elements identified by XRF (Ti, Al, Mo, Fe, Si) in corresponding regions. However, due to the limitations of EDS, especially in detecting light elements such as carbon and oxygen, XRF results are considered more reliable for bulk composition. The EDS method of chemical analysis identified such a basic alloy element: Ti 49.1 ± 0.7 wt.%, Al 26.6 ± 0.4 wt.%, and Mo 12.2 ± 0.3 wt.%. EDS analysis confirmed the higher carbon content of 7.3 ± 1.0 wt.%, which originates from the aluminothermic melting process aimed at obtaining hard–melting metal carbides. The alloy also contains oxygen 2.5 ± 0.8 wt.% due to the presence of hard and brittle Al_2_O_3_ particles and Si 1.4 ± 0.1 wt.%, which is contained mainly in the eutectic structure.

The results of both methods (XRF and EDS analysis) show that the alloy Ti55Al27Mo13 consists of the basic elements, namely titanium (approx. 55 wt.%), aluminium (approx. 27 wt.%), molybdenum (approx. 13 wt.%). In terms of the raw materials or the technology used to prepare the alloy, they are mainly present in the alloy: carbon (approx. 8 wt.%), silicon (approx. 2 wt.%), iron (approx. 1 wt.%), and also manganese (approx. 0.30 wt.%). Thus, we can state that the creation of a polycomponent alloy was achieved, where, in addition to the base metals (Ti, Al, Mo), cobalt and other elements (C, Si, Fe, Mn) are present in percentage units.

### 3.2. Microstructure of the Produced Alloy Ti55Al27Mo13

**Figure 6:**
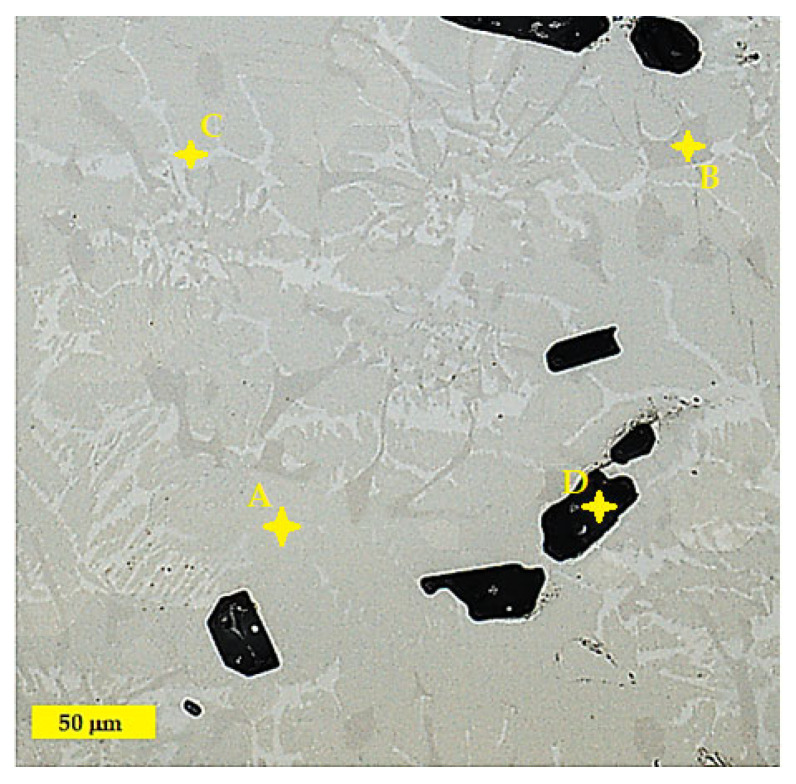
Ti55Al27Mo13 alloy microstructure with different structural phases: A—solid solution, B—solid solution, C—eutectic, and D—particles.

**Figure 7:**
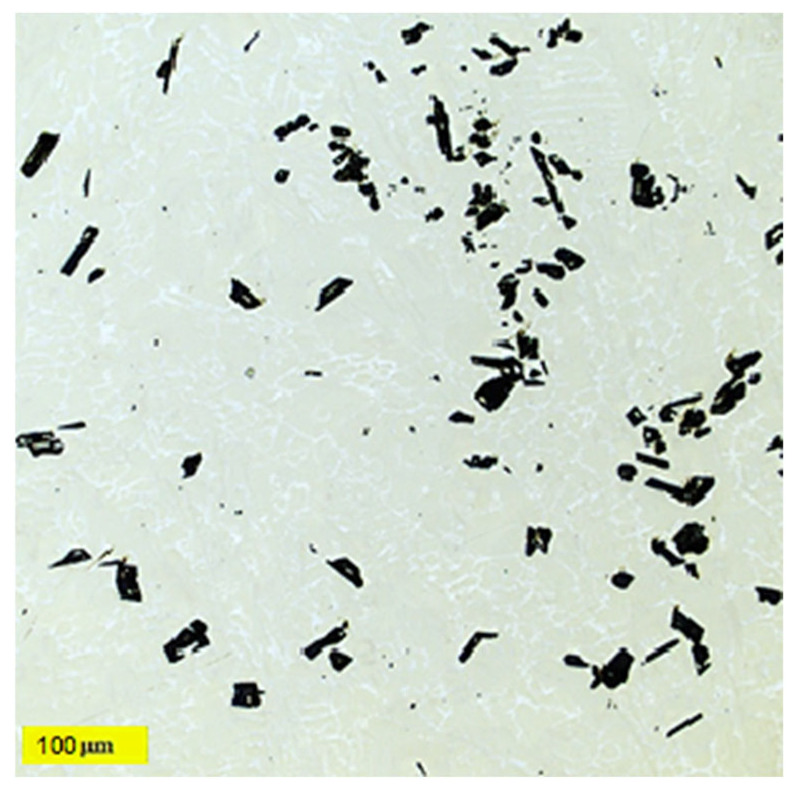
Ti55Al27Mo13 alloy microstructure with the dark particle’s local conglomeration.

The analysis and identification of individual structural components in the Ti55Al27Mo13 alloy were carried out on a metallographic cut using an OLS 3000 laser confocal microscope. The microstructure of the Ti55Al27Mo13 alloy consists of three different structural components: solid solutions, eutectic, and dark particles. The dark, coarse particles of irregular shape have a size range of 20 to 70 μm (labelled D in Figure 6). These types of particles exhibit an inhomogeneous distribution in their structure and form local conglomerates (see Figure 7). The first solid solution (labelled A in Figure 6) is light in colour, and the composition of this solid solution contains the basic elements Ti–Al–Mo. The darker solid solution (labelled B in Figure 6) is the second solid solution, and it has a different composition containing mainly Si. The structure of the experimental alloy contains the eutectic Ti–Al–Fe–Mo (labelled C in Figure 6). Figure 6 represents the distribution and frequency of dark particles in the structure, to see whether these particles are evenly distributed throughout the entire volume of the material, or whether they are agglomerations occurring only in certain places. This microscopic analysis revealed that the particles are distributed evenly throughout the material, but agglomerations also occur. From the perspective of surface wear, this constellation is advantageous. However, the mechanical properties will be lower due to agglomerations, as there is a presumption of stress concentration.

**Figure 8:**
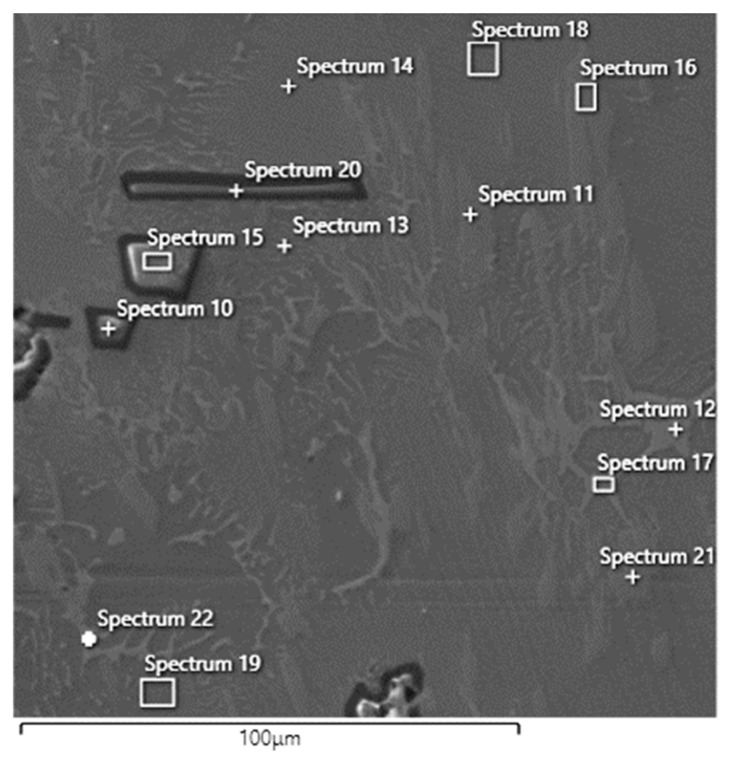
Ti55Al27Mo13 alloy EDS area analysis (Spectra 10–22, area 2 Figure 3).

**Figure 9:**
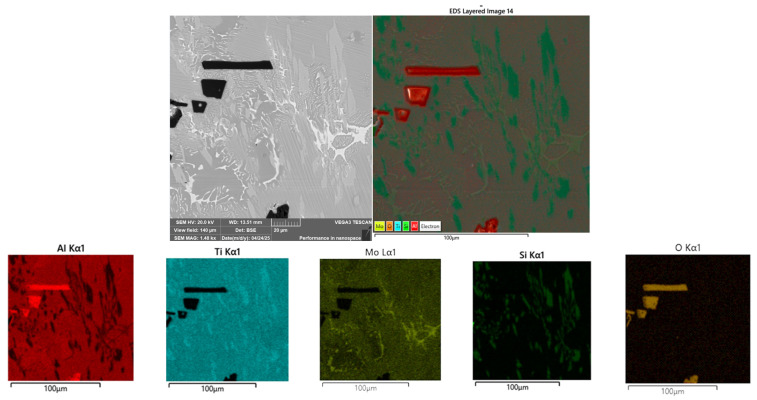
Elemental mapping of the Ti55Al27Mo13 alloy showing the distribution of titanium (Ti), aluminium (Al), molybdenum (Mo), oxygen (O), silicon (Si), and carbon (C), area 2 Figure 3.

The Tescan VEGA 3XMU scanning electron microscope, equipped with the Oxford EDS analyser, was used for a more detailed microstructure study and individual structural phase description (see Figure 8). Solid solutions A and B, the white eutectic structure, and dark irregular particles were determined using area analysis (see results in Table 3) and elemental mapping. All compositions are reported in [wt.%]. The elemental mapping visualised the distribution of the constituent elements in the specimen (Figure 9). This analysis revealed a relatively uniform distribution of elements in the alloy except for dark particles that are significantly different and contain a higher concentration of Al and O.

The area analysis of the eutectic structure (spectra 12, 13, 19, 17, 19, 22) revealed a composition based on titanium (on average 43.52 ± 3.0%), aluminium (20.56 ± 4.8%), molybdenum (20.18 ± 6.3%), and a small amount of iron (1.78 ± 1.3%). This structural component also contains a higher amount of carbon (8.62 ± 0.70%). Area and point EDS analyses of the two solid solutions (labelled A and B in Figure 6) revealed distinct differences in chemical composition and carbon content. Solid solution A (spectrum 14, 18) was composed primarily of titanium (48.2 ± 3.1 wt.%), aluminium (31.95 ± 2.2 wt.%), and molybdenum (13.05 ± 0.7 wt.%), with no detectable carbon. The homogeneity and composition suggest a Ti–Al–Mo–based phase with no secondary carbide formation. Its morphology is homogeneous and lighter in contrast under SEM. Structurally, it is considered a primary α–Ti (hcp) or TiAl–based solid solution, free of secondary precipitates. In contrast, solid solution B (spectrum 16, 11, and 21) exhibited a different chemical profile, containing titanium (56.93 ± 0.6 wt.%), aluminium (8.93 ± 0.2 wt.%), molybdenum (10.96 ± 0.04 wt.%), silicon (12.03 ± 0.1 wt.%), and a significant amount of carbon (8.76 ± 0.8 wt.%). Based on this composition, microstructural features, and enhanced microhardness values, we interpret this phase as a TiAl–based solid solution containing finely dispersed titanium carbide (TiC) particles. The carbon likely originates from the aluminothermic reaction involving powdered coal. The dispersed carbides contribute to the markedly higher hardness of this region, confirming their presence and mechanical relevance. It appears darker in SEM images and exhibits fine internal contrast differences, indicating the presence of finely dispersed TiC precipitates. The structure corresponds to a β–Ti (bcc) solid solution matrix with carbide strengthening. The increased hardness in this region supports this structural interpretation. The dark, sharp–edged particles of irregular shape (in some places, even partially regular geometric, sharp–edged particles; spectrum 10, 15, 20) range in size from 20 to 70 μm. The EDS analysis shows that the basis is oxygen (53.46 ± 0.1%) and aluminium (44.9 ± 1.6%). There is no carbon in these particles, and a minimal amount of titanium (1.6 ± 1.4%) is present. By calculating the stoichiometric ratio, we concluded that these are likely Al_2_O_3_ oxide particles. However, the stoichiometric ratio is slightly shifted in favour of oxygen, which may be caused by measurement inaccuracy or surface oxidation of the sample. Aluminium oxide is formed as a by–product of the aluminothermic reaction and is thermally very stable; it does not dissolve in the Ti–Al–Mo matrix even at high temperatures. When the melt solidifies, it is therefore precipitated as a separate ceramic phase. It is not a contaminant, but a natural product of the aluminothermic reaction, which can be “trapped” in the alloy structure. Their presence can lead to degradation of mechanical properties, particularly a decrease in the toughness and fatigue resistance of the alloy. On the other hand, when using this alloy in powder form to prepare resistant coatings, Al_2_O_3_ can contribute to wear resistance. The aluminothermic reaction, as described above, is the reduction of metal oxides by aluminium at high temperatures, with the by–product being aluminium oxide (Al_2_O_3_).

**Table 3:** Area EDS (Figure 8) analysis result with the individual elements’ concentration of the Ti55Al27Mo13 alloy.

Element	wt.% ± 3σ												
	Spectrum 10	Spectrum 11	Spectrum 12	Spectrum 13	Spectrum 14	Spectrum 15	Spektrum 16	Spectrum 17	Spectrum 18	Spectrum 19	Spectrum 20	Spectrum 21	Spectrum 22
Titanium	3.6 ± 0.1	57.4 ± 0.7	40.8 ± 0.5	47.2 ± 0.6	51.3 ± 0.3	0.7 ± 0.0	56.0 ± 0.7	41.0 ± 0.5	45.1 ± 0.6	47.3 ± 0.6	0.5 ± 0.0	57.4 ± 0.7	41.3 ± 0.5
Aluminium	42.6 ± 0.2	9.3 ± 0.2	20.2 ± 0.3	30.3 ± 0.4	34.2 ± 0.2	45.7 ± 0.2	8.9 ± 0.2	20.9 ± 0.3	29.7 ± 0.4	30.7 ± 0.4	46.4 ± 0.2	8.6 ± 0.1	20.7 ± 0.3
Molybdenum	0	11.0 ± 0.4	27.3 ± 0.4	14.0 ± 0.3	13.8 ± 0.3	0	10.9 ± 0.4	26.9 ± 0.5	12.3 ± 0.3	12.0 ± 0.3	0	11.0 ± 0.4	23.3 ± 0.4
Silicon	0	12.2 ± 0.2	0.4 ± 0.1	0	0.3 ± 0.1	0	11.9 ± 0.2	0.4 ± 0.1	0.4 ± 0.1	0.3 ± 0.1	0	12.0 ± 0.2	0
Carbon	0	8.1 ± 1.1	9.5 ± 1.0	7.3 ± 1.0	0	0	9.9 ± 1.0	9.3 ± 1.0	8.5 ± 0.9	8.5 ± 1.0	0	8.3 ± 1.1	8.5 ± 1.0
Oxygen	53.6 ± 0.2	0	0	0	0	53.6 ± 0.2	0	0	3.3 ± 0.6	0	53.2 ± 0.2	0	0
Iron	0	0	1.4 ± 0.1	0.9 ± 0.1	0.4 ± 0.1	0	0	1.1 ± 0.1	0.7 ± 0.1	1.0 ± 0.1	0	0	4.5 ± 0.1
Manganese	0	0	0.4 ± 0.1	0.4 ± 0.1	0	0	0	0.4 ± 0.1	0	0.3 ± 0.1	0	0	0.9 ± 0.1
Copper	0	0	0	0	0	0	0	0	0	0	0	0	0.9 ± 0.1
Phosphorus	0	2.1 ± 0.1	0	0	0	0	2.3 ± 0.1	0	0	0	0	2.7 ± 0.1	0
Sulphur	0.2 ± 0.0	0	0	0	0	0	0	0	0	0	0	0	0

The elemental mapping of the Ti55Al27Mo13 alloy (Figure 9) reveals essential information about the spatial distribution of constituent elements. Titanium, aluminium, and molybdenum are distributed relatively homogeneously throughout the matrix, which corresponds to the base Ti–Al–Mo solid solution phases. Notably, oxygen and aluminium are intensely concentrated in the irregular dark particles, confirming their identification as aluminium oxide (Al_2_O_3_). These oxide phases are a natural by–product of the aluminothermic reaction and remain undissolved in the metal matrix. The mapping also shows localised regions with elevated concentrations of carbon and silicon, which correlate with solid solution B, where finely dispersed TiC particles are assumed to form. This observation supports the microstructural interpretation derived from point EDS and hardness measurements.

**Figure 10:**
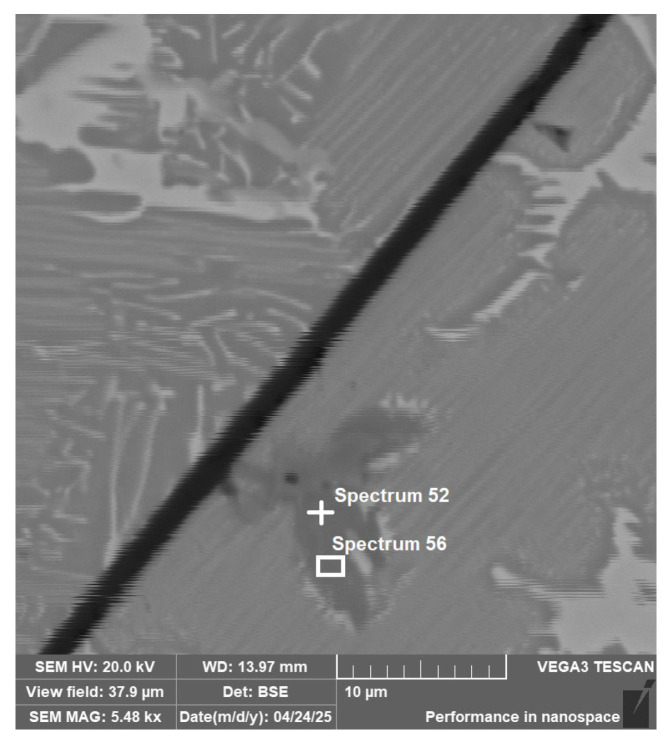
Ti55Al27Mo13 alloy EDS area and point analysis (area 3 Figure 3).

The separate titanium phases also occur in the alloy Ti55Al27Mo13. The results of the EDS analysis from Figure 10 are presented in Table 4. By stoichiometric calculation of the element content in the spectrum, it was found that the element ratio corresponds to approximately Ti_2_AlC_2_. This corresponds to the composition of MAX phases, such as Ti_2_AlC, a known ceramic phase with excellent mechanical properties.

**Table 4:** EDS (Figure 10) analysis result with the individual elements concentration of the Ti55Al27Mo13 alloy.

Element	wt.% ± 3σ	
	Spectrum 52	Spectrum 56
Titanium	66.3 ± 0.6	65.9 ± 0.5
Aluminium	17.3 ± 0.2	19.5 ± 0.2
Molybdenum	0.5 ± 0.2	0.4 ± 0.1
Carbon	15.9 ± 0.8	14.2 ± 0.6

### 3.3. Mechanical Properties of the Produced Alloy Ti55Al27Mo13

To evaluate the mechanical behaviour of the Ti55Al27Mo13 alloy at different structural scales, we used two hardness testing methods. Rockwell C hardness was measured on the bulk–polished sample to characterise the general hardness of the entire alloy. In parallel, micro–Vickers hardness testing (HV 0.01) was performed on individual microstructural constituents in metallographic sections to assess the contribution of each phase to the alloy’s mechanical response. This dual approach enables the correlation of phase composition and morphology with localised hardness values, supporting the understanding of structure–property relationships in this heterogeneous alloy system.

The average Rockwell hardness C of the experimental alloy Ti55Al27Mo13 is 72.28 ± 5.59 HRC.

We measured the lowest Vickers hardness in the eutectic structure 860.3 ± 32.7 HV 0.01. Solid solutions A and B have different hardness. The lowest hardness was exhibited by solid solution A, with a hardness value of 934.4 ± 118.7 HV 0.01. Solid solution B had a hardness of 1364.8 ± 175.3 HV 0.01. The dark particles reached an average hardness of 2411.2 ± 347.4 HV 0.01. As can be seen from the measurements, the standard deviation takes on relatively high values. This is due to particle heterogeneity in the structure, structural inhomogeneity, or differences between individual grains, especially when the grains are large relative to the imprint in the indentation.

## 4. Discussion

The Ti55Al27Mo13 alloy composition lies within the Ti–rich and Mo–containing region of the ternary diagram, suggesting the possible coexistence of β(Ti,Mo), TiAl, and potentially the ρ–phase or Ti_3_Al, depending on processing conditions. The alloy also contains carbon, silicon, and oxide particles (Al_2_O_3_) due to the aluminothermic process. These factors affect the microstructure and mechanical performance.

### 4.1. Comparison of Prepared Ti55Al27Mo13 Alloy Composition by XRF and EDS Analysis

Table 5 shows a comparison of the alloy Ti55Al27Mo13 XRF chemical composition and area EDS analysis.

The results of the comparison demonstrate that there is little difference between the two methods. Using EDS analysis, it is possible to determine carbon content, which was not possible with XRF analysis. In addition to bulk chemical analysis (Table 2), local compositional differences between microstructural features were evaluated using EDS. This method provided spatially resolved information on the distribution of elements within different phases (Table 3), including oxide inclusions, eutectic areas, and carbide–rich regions, which could not be captured by bulk XRF analysis.

Table 6 demonstrates the individual Ti55Al27Mo13 alloy phases, along with their respective chemical compositions and mechanical properties (Vickers microhardness). The dark particles achieve the highest hardness. Phases with the lowest hardness have a eutectic structure, and from the chemical point of view, they contain the most iron. Solid solution A and B differ by about 430 HV 0.01, while the more complex solid solution B contains more titanium, silicon, and carbon. The measured hardness of these dark particles (~2411 HV) is consistent with values reported for α–Al_2_O_3_ (corundum) in the literature, which typically range between 2000 and 2300 HV for dense, crystalline alumina [36,37]. Their chemical composition (dominance of Al and O with minimal Ti), morphology, and separation behaviour during solidification are in agreement with alumina inclusions commonly formed in aluminothermic reactions. The near absence of titanium excludes the formation of ternary oxides such as TiAlO, which are not stable bulk phases and exhibit significantly lower hardness. The differences in hardness between solid solutions A and B can be directly correlated with their chemical and structural characteristics. Solid solution A, with no detectable carbon and lower silicon content, represents a typical Ti–Al–Mo solid solution phase, likely with an α–Ti or TiAl matrix, and exhibits moderate hardness (~930 HV). Solid solution B, on the other hand, contains significant amounts of carbon and silicon, which result in the formation of finely dispersed TiC carbides within a TiAl–based matrix. These carbides act as substantial obstacles to dislocation motion, providing effective dispersion strengthening. In addition, the presence of silicon may also contribute to solution strengthening. The combined effects of TiC precipitation and solid solution hardening explain the increased microhardness of solid solution B (~1365 HV), in agreement with similar observations reported in TiC–reinforced titanium alloys [38,39,40,41].

### 4.2. Comparison of Prepared Ti55Al27Mo13 Alloy with Other Special Titanium Alloys

To strengthen the connection between composition and microstructure, the following observations were made. The high Mo content (13 wt.%) is associated with the formation of a Mo–rich solid solution B, as identified via EDS and confirmed by hardness mapping. These regions exhibited microhardness values of approximately 1300 HV, significantly higher than those of the matrix, which supports the well–established role of Mo as a solid solution–strengthening and β–stabilising element. In addition, minor Si enrichment (up to ~0.8 wt.%) was detected in the same areas, often in conjunction with carbon. This suggests the possible formation of thermally stable (Mo, Si)–rich carbides or silicides, which are also known to improve oxidation resistance. These localised chemical effects are reflected in the microstructural contrast and contribute to the mechanical heterogeneity of the alloy.

Compared to conventional titanium alloys such as Ti–6Al–4V, Ti–6242, or Ti–15Mo, the Ti55Al27Mo13 alloy offers unique advantages in terms of surface hardness and thermal stability due to its heterogeneous microstructure. The formation of challenging phases—such as Al_2_O_3_, TiC, and possibly Ti_2_AlC—enhances its resistance to abrasion and high–temperature oxidation. The average matrix hardness (~900 HV) and peak values exceeding 2400 HV in oxide regions are significantly higher than those of typical α + β or β titanium alloys (usually <400 HV). These properties make the material especially suitable for protective coatings rather than bulk structural use. Moreover, its preparation via aluminothermic reaction allows for the incorporation of hard–to–melt elements and ceramic phases in a single step, presenting an efficient and cost–effective alternative to conventional melting and powder metallurgy techniques.

Although Ti–Al–Mo alloys are known for their high–temperature strength and oxidation resistance, the specific Ti55Al27Mo13 composition developed in this work is novel in terms of its synthesis route and resulting phase structure. The alloy was prepared by aluminothermic reduction, which enabled the direct formation of a complex, multi–phase microstructure, including challenging ceramic phases, in a single high–temperature step. This approach differs significantly from conventional melting or powder metallurgy techniques applied to similar compositions.

In summary, the Ti55Al27Mo13 alloy represents a promising candidate for protective surface technologies, especially where extreme hardness, thermal durability, and resistance to oxidative degradation are required. Its use as a precursor for fine–grained composite powders opens the door for advanced thermal spray, laser cladding, or sintering applications in the aerospace, energy, and automotive industries.

The Ti55Al27Mo13 alloy composition lies within the Ti–rich and Mo–containing region of the ternary diagram, suggesting the possible coexistence of β(Ti,Mo), TiAl, and potentially the ρ–phase or Ti_3_Al, depending on processing conditions. Understanding these phase equilibria is therefore essential for interpreting the microstructure, thermal stability, and mechanical behaviour of aluminothermically prepared Ti–Al–Mo alloys. The produced Ti55Al27Mo13 alloy contains an admixture of carbon, iron, and silicon and small amounts of manganese, copper, and cobalt. Table 7 shows a comparison of the Ti55Al27Mo13 prepared by aluminothermic reaction with special titanium alloys with Mo content in terms of structure, high–temperature behaviour, hardness, and production technology [36].

Compared to conventional titanium alloys such as Ti–6Al–4V, Ti–6242, or Ti–15Mo, the Ti55Al27Mo13 alloy offers unique advantages in terms of surface hardness and thermal stability due to its heterogeneous microstructure. For the formation of challenging phases—such as Al_2_O_3_, TiC, and possibly Ti_2_AlC—we assume their resistance to abrasion and oxidation at high temperatures. The average matrix hardness (~900 HV) and peak values exceeding 2400 HV in oxide regions are significantly higher than those of typical α + β or β titanium alloys (usually <400 HV). These properties make the material especially suitable for protective coatings rather than bulk structural use. Moreover, its preparation via aluminothermic reaction allows for the incorporation of hard–to–melt elements and ceramic phases in a single step, presenting an efficient and cost–effective alternative to conventional melting and powder metallurgy techniques.

As shown in Table 7, the Ti55Al27Mo13 alloy offers several notable advantages compared to conventional titanium alloys such as Ti–6242, Ti–1100, and Ti–15Mo. While those alloys typically exhibit hardness values between 220 and 400 HV, the Ti55Al27Mo13 alloy demonstrates significantly higher hardness, both in the matrix (~930–1365 HV) and especially in the Al_2_O_3_ ceramic inclusions (~2400 HV). This high hardness suggests strong potential for wear resistance in demanding environments.

Moreover, the presence of dispersed ceramic (Al_2_O_3_) and carbide (TiC) phases formed in situ during the aluminothermic reaction provides enhanced microstructural stability at elevated temperatures. The alloy also benefits from an estimated lower production cost, as the aluminothermic route does not require high–vacuum melting or precision alloying.

In summary, the Ti55Al27Mo13 alloy represents a promising candidate for protective surface technologies, especially where extreme hardness, thermal durability, and resistance to oxidative degradation are required. Its use as a precursor for fine–grained composite powders opens the door for advanced thermal spray, laser cladding, or sintering applications in the aerospace, energy, and automotive industries. While further testing is needed to confirm properties such as fatigue life and oxidation resistance, the current results position Ti55Al27Mo13 as a promising candidate for applications where hardness, potential wear resistance, and process efficiency are key factors.

## 5. Conclusions

In this article, we present the results of a new alloy that we successfully prepared by an aluminothermic reaction. This alloy will be used for the subsequent preparation of a special composite nanopowder and surface coating of aluminium, magnesium, and iron alloys. The results obtained can be summarised in the following points.

(1) The XRF results of the alloy chemical analysis showed that the alloy is composed of titanium 54.86 wt.%, aluminium 27.34 wt.%, molybdenum 13.62 wt.%, and small amounts of silicon and iron from the aluminothermic reaction and other components (Mn, Cu, Co, Nb).
(2) Elements such as C and O were identified in more detail using EDS analysis.
(3) The microstructure was characterised using laser and scanning electron microscopy. The microstructure consists of several structural components, namely solid solution A, solid solution B, eutectic, and particles ranging from 20 to 70 micrometres in size and with sharp, irregular edges. Solid solution A is composed of titanium, aluminium, and molybdenum; in solid solution B, the predominant element is silicon and carbon. The eutectic has a higher proportion of molybdenum and iron and the particles were identified as Al_2_O_3_. Separate phases of the Ti_2_AlC type with a high carbon content also occur in the structure.
(4) The presence of Al_2_O_3_ particles in the Ti55Al27Mo13 alloy prepared by aluminothermic reaction can be explained as a result of secondary oxidation of aluminium, which serves as a reducing agent in the response. The resulting aluminium oxide (Al_2_O_3_) is thermodynamically very stable and does not dissolve in the metal matrix. Therefore, it occurs as a separate ceramic phase in the resulting microstructure. Its morphology (sharp–edged particles and needles 20–70 μm in size) corresponds to the growth of corundum crystals under conditions of slow cooling and sufficient time for crystallisation.
(5) The use of both Rockwell C and micro–Vickers hardness methods provides complementary insights into the mechanical behaviour of the Ti55Al27Mo13 alloy. While the Rockwell C test gives an integral view of the overall hardness of the alloy as a bulk material (72.3 ± 5.6 HRC), the micro–Vickers results distinguish the mechanical contributions of individual structural components. For example, the high hardness values of the Al_2_O_3_ particles (~2400 HV 0.01) explain the increased average bulk hardness, while the differences between solid solution A (~930 HV) and B (~1365 HV) reflect the influence of carbon and silicon content on phase strengthening. This multiscale approach confirms the correlation between phase composition, microstructure, and mechanical performance, which is crucial for applications that require both wear resistance and structural integrity.

This research brought essential insights into the creation of new Ti–Al–Mo alloys. It revealed the possibility of converting high–melting metals (Ti, Mo, Si) into corresponding structural components using an aluminothermic reaction, the beneficial by–product of which is the formation of ceramic, highly hard aluminium oxide particles, the properties of which will primarily be utilised to enhance the wear resistance of the alloy. These insights not only aid in characterising the phases present in the Ti55Al27Mo13 alloy but also provide a thermodynamic foundation for further alloy design and optimization of processing within the Ti–Al–Mo system.

## Figures and Tables

**Table 5:** Chemical composition results of the produced Ti55Al27Mo13 alloy analysed by XRF and area EDS analysis.

Chemical Element [wt.%]	Average (XRF)	Average (EDS)
Ti	54.86	49.1
Al	27.34	26.6
Mo	13.62	12.2
Fe	0.99	0.8
Si	2.14	1.4
Mn	0.28	–
Cu	0.32	–
Co	0.16	–
Nb	0.038	–
C	–	7.3
O	–	2.5

**Table 6:** Results of the produced alloy Ti55Al27Mo13 phase composition analysed by EDS analysis and Vickers microhardness HV 0.01.

Phase	Chemical Composition, EDS [wt.%]		HV 0.01
Solid solution A	Ti	48.2 ± 3.1	934.4
	Al	31.95 ± 2.2	
	Mo	13.05 ± 0.7	
Solid solution B	Ti	56.93 ± 0.6	1364.8
	Al	8.93 ± 0.2	
	Mo	10.96 ± 0.04	
	Si	12.03 ± 0.1	
	C	8.76 ± 0.8	
Eutectic	Ti	43.52 ± 3.0	860.3
	Al	20.56 ± 4.8	
	Mo	20.18 ± 6.3	
	Fe	1.78 ± 1.3	
	C	8.62 ± 0.70	
Particles	O	53.46 ± 0.1	2411.2
	Al	44.9 ± 1.6	
	Ti	1.6 ± 1.4	

**Table 7:** Comparison of Ti55Al27Mo13 with the special titanium alloys with Mo content.

Parameter/Alloy	Ti55Al27Mo13	Ti–6242 (Ti–6Al–2Sn–4Zr–2Mo) [41,42]	Ti–1100 [43,44]	Ti–15Mo [45,46]
Composition [wt.%]	Ti–55Al–27Mo (+Al_2_O_3_)	Ti–6Al–2Sn–4Zr–2Mo	Ti–6Al–2.8Sn–4Zr–6Mo	Ti–15Mo
Alloy type	Intermetallic with dispersed phase	α + β high–temperature	α + β	β (metastable)
Hardness HV	900 (matrix), Al_2_O_3_ 2400	340–380	350–400	220–280
Max. operating temperature [°C]	–	540–590	590–600	~350–450
Production technology	aluminothermy	conventional melting	conventional melting	powder metallurgy, formed

## Data Availability

The original contributions presented in this study are included in the article. Further inquiries can be directed to the corresponding author.

## References

[1] Michna Š., Knaislová A., Hren I., Novotný J., Michnová L., Svobodová J. (2022). Chemical and Structural Analysis of Newly Prepared Co–W–Al Alloy by Aluminothermic Reaction. Materials.

[2] Maleki A., Hosseini N., Niroumand B. (2018). A review on aluminothermic reaction of Al/ZnO system. Ceram. Int..

[3] Xing Z., Lu J., Ji X. (2018). A Brief Review of Metallothermic Reduction Reactions for Materials Preparation. Small Methods.

[4] Gostishchev V.V., Kim E.D., Khimukhin S.N., Ri E.H. (2019). High–Temperature Synthesis of Al–Zr–W Aluminum–Matrix Alloys. Inorg. Mater..

[5] Kirakosyan H., Nazaretyan K., Kharatyan A., Aydinyan S. (2024). The preparation of high–entropy refractory alloys by aluminothermic reduction process. AIP Conf. Proc..

[6] Zhang W., Chabok A., Kooi B.J., Pei Y. (2022). Additive manufactured high entropy alloys: A review of the microstructure and properties. Mater. Des..

[7] Birol Y. (2012). Aluminothermic reduction of boron oxide for the manufacture of Al–B alloys. Mater. Chem. Phys..

[8] Meir Y., Jerby E. (2015). Underwater microwave ignition of hydrophobic thermite powder enabled by the bubble–marble effect. Appl. Phys. Lett..

[9] de Souza K.M., de Lemos M.J.S. (2021). Detailed Numerical Modeling and Simulation of Fe_2_O_3_−Al Thermite Reaction. Propellants Explos. Pyrotech..

[10] Koch E.C., Knapp S. (2019). Thermites—Versatile Materials. Propellants Explos. Pyrotech..

[11] Kim D.K., Bae J.H., Kang M.K., Kim H.J. (2011). Analysis on thermite reactions of CuO nanowires and nanopowders coated with Al. Curr. Appl. Phys. Juneec.

[12] Moore J.J., Feng H.J. (1995). Combustion synthesis of advanced materials: Part, I. Reaction parameters. Prog. Mater. Sci..

[13] Manojlovic V., Kamberovic Ž., Gavrilovski M., Sokic M., Korac M. (2017). Combustion of Metallurgical Wastes Using Secondary Aluminum Foils. Combust. Sci. Technol..

[14] Cojocaru M., Branzei M., Coman T.A. (2015). Thermodynamics of Iron Metallothermy. Adv. Mater. Res..

[15] United States Military Chemistry and Chemical Agents. https://catalog.hathitrust.org/Record/009425398.

[16] Venugopalan R., Sathiyamoorthy D. (2006). Investigation through factorial design on novel method of preparing vanadium carbide using carbon during aluminothermic reduction. J. Mater. Process. Technol..

[17] Biswas A., Nair K.U., Bose D.K. (1993). Preparation of single–phase Cr_7_C_3_ by aluminothermic reduction. J. Alloys Compd..

[18] Xu Y., Yang Z., Han Z., Liu G., Li J. (2014). Fabrication of Ni/WC composite with two distinct layers through centrifugal infiltration combined with a thermite reaction. Ceram. Int..

[19] Sheybani K., Paydar M.H., Shariat M.H. (2019). Effect of mechanical activation on aluminothermic reduction of molybdenum trioxide. Int. J. Refract. Met. Hard Mater..

[20] Distl B., Stein F. (2024). Ti–Al–based alloys with Mo: High–temperature phase equilibria and microstructures in the ternary system. Philos. Mag..

[21] Singh A.K., Banumathy S., Sowjanya D., Rao M.H. (2008). On the structure of the B2 phase in Ti–Al–Mo alloys. J. Appl. Phys..

[22] Chen Z., Jones I.P., Small C.J. (1997). The structure of the alloy Ti 50Al 15Mo between 800 °C and 1400 °C. Acta Mater..

[23] Abdoshahi N., Dehghani M., Hatzenbichler L., Spoerk–Erdely P., Ruban A.V., Musi M., Mayer S., Spitaler J., Holec D. (2021). Structural stability and mechanical properties of TiAl+Mo alloys: A comprehensive *ab initio* study. Acta Mater..

[24] Huang X.M., Zhu L.L., Cai G.M., Liu H.S., Jin Z.P. (2017). Experimental investigation of phase equilibria in the Ti–Al–Mo ternary system. J. Mater. Sci..

[25] Kulkarni K. Investigation of Interdiffusion and Diffusional Interactions in the Ternary Ti–Al–Mo Alloys. Master’s Thesis.

[26] Yang G., Xu X., Liang Y., Wang Y., Hao G., Zhai Y., Lin J. (2021). Effects of Al and Mo on Microstructure and Hardness of As–Cast TNM TiAl Alloys. Metals.

[27] Tang J.J., Liang C., Xu C.G., Li J.Q. (2022). Effect of Alloying Elements on Strengthening Phase and Solidification Structure of Ti–Al–Mo–Zr Titanium Alloy. Adv. Mater. Res..

[28] Singh A.K., Banerjee D. (1997). Transformations in α2+γ titanium aluminide alloys containing molybdenum: Part, I. Solidification behavior. Metall. Mater. Trans. A.

[29] Azad S., Mandal R.K., Singh A.K. (2006). Effect of Mo addition on transformation behavior of (α2 + γ) based Ti–Al alloys. Mater. Sci. Eng. A..

[30] Gupta J., Ghosh S., Aravindan S. (2021). Effect of Mo content on Ti–Al–Mo ternary alloys for biomedical applications. Mater. Lett..

[31] Sun C., Xiao R., Li H., Ruan Y. (2022). Effects of phase selection and microsegregation on corrosion behaviors of Ti–Al–Mo alloys. Corros. Sci..

[32] Jimenez–Marcos C., Mirza–Rosca J.C., Baltatu M.S., Vizureanu P. (2023). Effect of Si Contents on the Properties of Ti15Mo7ZrxSi Alloys. Materials.

[33] Jiang Z., Dai X., Middleton H. (2011). Effect of silicon on corrosion resistance of Ti–Si alloys. Mater. Sci. Eng. B..

[34] Kaur M., Singh K. (2019). Review on titanium and titanium based alloys as biomaterials for orthopaedic applications. Mater. Sci. Eng. C..

[35] Jirón–Lazos U., Rodil S.E., Mazón–Montijo D.A., Pérez–Higareda J.R., Torres–Torres D., Garay–Tapia A.M., Montiel–González Z. (2023). Microstructural behavior of the Ti–Al–Mo–N system controlled by Mo content: Impact on the performance as hard coatings. J. Mater. Sci..

[36] Leyens C., Peters M. (2003). Titanium and Titanium Alloys: Fundamentals and Applications.

[37] Xu X., Zhang Q., Wu J., Wang H., Tian K., Wu C. (2021). Preparation and characterization of corundum–based ceramics for thermal storage. Ceram. Int..

[38] Wang N., Choi Y., Matsugi K. (2023). Effect of C content on the microstructure and properties of in–situ synthesized TiC particles reinforced Ti composites. Sci. Rep..

[39] Wang Z., Cheng H., Lv Y., Zhang Z., Fan J., Zhang H., Liu B., Ma Z. (2022). Effect of TiC content on the microstructure and mechanical properties of Ti–30Mo–xTiC composites. Int. J. Refract. Met. Hard Mater..

[40] Wei W.H., Shao Z.N., Shen J., Duan X.M. (2018). Microstructure and mechanical properties of in situ formed TiC–reinforced Ti–6Al–4V matrix composites. Mater. Sci. Technol..

[41] Fargas G., Roa J.J., Sefer B., Pederson R., Antti M.L., Mateo A. (2018). Influence of cyclic thermal treatments on the oxidation behavior of Ti–6Al–2Sn–4Zr–2Mo alloy. Mater. Charact..

[42] Semiatin S.L., Thomas J.F., Dadras P. (1983). Processing–microstructure relationships for Ti–6Al–2Sn–4Zr–2Mo–0.1Si. Metall. Trans. A.

[43] Stella P., Giovanetti I., Masi G., Leoni M., Molinari A. (2013). Microstructure and microhardness of heat–treated Ti–6Al–2Sn–4Zr–6Mo alloy. J. Alloys Compd..

[44] Carrozza A., Aversa A., Fino P., Lombardi M. (2021). A study on the microstructure and mechanical properties of the Ti–6Al–2Sn–4Zr–6Mo alloy produced via Laser Powder Bed Fusion. J. Alloys Compd..

[45] Gatina S.A., Polyakova V.V., Polyakov A.V., Semenova I.P. (2022). Microstructure and Mechanical Properties of β–Titanium Ti–15Mo Alloy Produced by Combined Processing including ECAP–Conform and Drawing. Materials.

[46] Awad A.H., Aly H.A., Saood M. (2024). Physical, mechanical, and corrosion properties of Ti–12Mo and Ti–15Mo alloys fabricated by elemental blend and mechanical alloying techniques. Mater. Chem. Phys..

